# Effect of Physical Aging on Gas Transport in Asymmetric Polyimide Hollow Fibers Prepared by Triple-Orifice Spinneret

**DOI:** 10.3390/polym12020441

**Published:** 2020-02-13

**Authors:** Gabriele Clarizia, Franco Tasselli, Paola Bernardo

**Affiliations:** Institute on Membrane Technology (ITM-CNR), c/o University of Calabria, via P. Bucci 17/C, 87036 Rende (CS), Italy; g.clarizia@itm.cnr.it (G.C.); f.tasselli@itm.cnr.it (F.T.)

**Keywords:** Hollow-fiber membranes, physical aging, triple-orifice spinneret, crosslinking, gas separation

## Abstract

The systematic evaluation of the gas transport properties related to differences in the history of the samples is a useful tool to appropriately design a membrane-based gas separation system. The permeation rate of six pure gases was measured over time in asymmetric hollow-fiber (HF) samples, that were prepared according to the non-solvent-induced phase separation in different operation conditions, in order to identify their response to physical aging. Four types of HFs having a different structure were analyzed, comparing samples spun in a triple-orifice spinneret to HFs prepared using a conventional spinneret. A generalized gas permeance decline, coupled to a maintained permselectivity for the different gas pairs, was observed for all HFs. Instead, H_2_/N_2_ permselectivity values were enhanced upon aging. Cross-linked hollow-fiber samples showed a marked size-sieving behavior that favored the separation of small species, e.g., hydrogen, from other larger species and a good stability over time. Some HFs, post-treated using different alcohols, presented a permeance decay independently on the alcohol type and a greater selectivity over time.

## 1. Introduction

Membrane-based gas separation represents a promising alternative to conventional unit operations in chemical and process industries [1,2]. The development of membranes based on more permselective and stable materials is a fundamental challenge for enhancing the competitiveness of this environmentally friendly technology. 

Hollow fibers (HFs) are the dominant configuration in membrane gas separation since they result in more compact systems by virtue of the highest packing density [3]. In previous works, we proved the possibility of tailoring the properties of HFs, opportunely changing the preparation conditions while also using a triple-orifice spinneret and specific post-treatment protocols [4,5].

Since the membrane productivity is determined by the permeation rate of the species through the permselective film, the opportunity of enhancing the gas flux is of fundamental importance for wide-ranging applications of technological interest. Therefore, we also investigated the post-treatment of these membranes, demonstrating that a general protocol adopted in the literature for different membrane operations has to be customized according the specific morphology of the HFs [6].

The triple-orifice spinneret used to feed an external fluid during the air-gap traveling of the nascent fiber was proven to be an appropriate tool to modify the morphology and then the gas transport properties of HFs. Indeed, the beneficial effect of the external fluid, capable of increasing both gas permeance and permselectivity, was also demonstrated in combination with the in-line crosslinking.

Physical aging is a progressive and quite irreversible phenomenon that affects all polymeric glassy materials [7]. Due to its repercussions on large-scale systems, it represents one of the most significant problems to consider for the affirmation of this innovative technology. As a consequence, several studies were performed to investigate this phenomenon in more depth and how to limit its incidence.

Upon physical aging, the polymer free volume reduces, thus affecting different properties (e.g., mechanical and thermal properties) and depressing the gas transport rates [8]. These phenomena were reported for different materials, including polyimides (e.g., Matrimid), as free-standing films [7,9,10,11,12]. However, the effects occur with different rates depending on their specific morphology and mainly on sample thickness [13,14,15]. Typically, as the thickness of the film increases, the aging requires more time and it is delayed [16,17]. Therefore, thin films are subjected to a physical aging faster than thick ones. On free-standing polymer films, protocols of accelerated physical aging were carried out by several authors, bringing the samples for certain times above the glass transition temperature, deleting the history of the film preparation [18,19]. 

Different aging studies were done on hollow fibers, analyzing the effects of chemical structure on gas separation performance [20,21,22]. As pointed out by Koros et al. [9], physical aging of hollow fibers can behave differently from dense films. This is due to the rapid quenching during the spinning process that traps a large amount of free volume in the hollow fibers, while isotropic dense films prepared by solution casting are obtained after a slow solvent evaporation. Indeed, the nascent fiber extruded through the spinneret passes the air gap before entering into the coagulation bath, where phase separation occurs rapidly and the dope demixes into a polymer-rich phase and a polymer-lean phase that constitutes the pores. Furthermore, artificial accelerated physical aging procedures cannot be applied to hollow fibers without damaging their delicate supported asymmetric structures.

For these reasons, this work was mainly performed by monitoring, over time, the gas transport properties of asymmetric hollow fibers, based on Matrimid^®^, prepared in different conditions and eventually subjected to different post-treatments. The aging behavior was correlated to the HF morphology that can be tailored by selecting proper spinning conditions. A deeper knowledge of the aging on hollow fibers, characterized by different structures, makes the results of this study useful for a rational design of separation systems of industrial interest.

## 2. Materials and Methods

The polyimide, Matrimid^®^5218 (3,3′,4,4′-benzophenonetetracarboxylic dianhydride and diaminophenylindane), was provided by Huntsman Advanced Materials (Everberg, Belgium). Ethylene diamine (EDA), from Sigma Aldrich (Milan, Italy), was used as a crosslinker in aqueous solutions. 

*N*-Methyl-2-pyrrolidone (NMP, purity of 99%, VWR International, Milan, Italy) was used to dissolve the polyimide for the HF spinning. Ethanol (EtOH), butanol (ButOH), and *t*-butanol (*t*-ButOH), purchased from VWR International (Milan, Italy), were adopted for the solvent exchange procedure. All the chemicals were used as received.

A bi-component epoxy resin (Elan Tech^®^, supplied by ELANTAS Italia S.r.l., Ascoli Piceno, Italy) was used for potting the HFs within the modules used for gas permeation tests. The gases used in the permeation tests (CO_2_, He, H_2_, N_2_, O_2_, and CH_4_) were purchased from SAPIO (Monza, Italy) with a purity of 99.99%. 

### 2.1. Hollow-Fiber Spinning

**Figure 1:**
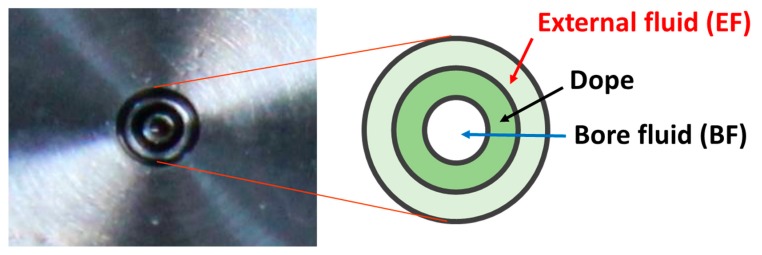
Scheme of the triple-orifice spinneret used for preparing the hollow fibers (HFs).

Different batches of HFs were spun, changing the composition of the bore fluid (BF) and the external fluid (EF). The polymer was dissolved in NMP at a concentration of 24 wt % at 50 °C. The dope solution was left under magnetic stirring overnight and, before the spinning, the dope was degassed under vacuum for a few minutes. A pilot plant described elsewhere was used for the spinning process [5]. The dope flow rate was 5 g∙min^−1^, while the EF and BF flow rates were 3 g∙min^−1^. The air gap was 60 cm. The triple-orifice spinneret, depicted in Figure 1, was described in Reference [4].

Table 1 reports the code assigned to each batch of HFs and the composition for the BF and the EF used for the spinning. Reference HFs (C1) were spun by feeding de-ionized water as bore fluid without the external fluid, thus using the triple-orifice spinneret as a conventional double orifice spinneret. Other HFs were prepared in the triple-orifice spinneret by feeding an external fluid through the spinneret on the outer side of the fiber, in the dry-jet step between the spinneret exit and the water bath, using water or a solvent-rich solution as BF (T1 and T2, respectively). The last HF type was obtained by simultaneously applying a crosslinking, feeding dilute EDA solutions in water as BF (T3) and a solvent-rich EF. Three batches were produced by changing the EDA concentration in the BF. The as-spun HFs were kept in de-ionized water at room temperature for two days to ensure complete solvent–nonsolvent exchange, followed by thorough rinsing with water. In some cases, the HFs were post-treated for a solvent exchange with baths of an alcohol (three baths of 20 min each). According to the systematic study on the effect of the post-treatment protocol [6], *n*-hexane was not used as final solvent since it typically resulted in extremely permeable membranes with a very poor permselectivity in gas separation. Finally, the HFs were dried in air in room conditions, and some representative samples were sealed in the modules for testing.

### 2.2. Morphological Analysis

The cross-section of the prepared HFs was observed using a scanning electron microscope (SEM EVO|MA 10, Zeiss, Milano, Italy). The HFs were fractured in liquid nitrogen and then sputter-coated with gold. Sample images were acquired in high-vacuum mode, working at 20 kV. 

### 2.3. Gas Permeation Tests

Gas permeation tests were carried out with single gases (H_2_, He, N_2_, O_2_, CH_4_, and CO_2_) in a fixed-volume instrument (Elektro & Elektronik Service Reuter, Geesthacht, Germany). A high-vacuum equipment, comprising a turbo molecular pump and a membrane backing pump, was used for the evacuation of the samples before each test. HF samples were potted in a short aluminum tube and sealed at the other end. The active HF length was approximately 10 cm. The permeation tests were carried out at a feed pressure of 1 bar and 25 °C, feeding the gas on the lumen side in dead-end mode. 

Each HF sample was exposed to the feed gas, while the pressure increase in the permeate volume, caused by the gas permeation through the membrane, was monitored by a pressure transducer over time. The pressure signal was correlated to the amount of gas permeated through the sample, as described in Reference [23], evaluating the gas permeance, expressed in gas permeance units (GPU; 1 GPU = 10^−6^ cm^3^ (standard temperature and pressure (STP))/(cm^2^∙s∙cm Hg)). The ideal permselectivity was calculated as the ratio of pure gas permeance coefficients.

When not subjected to experimental tests, the membranes were stored in air in room conditions (static aging).

## 3. Results

**Figure 2:**
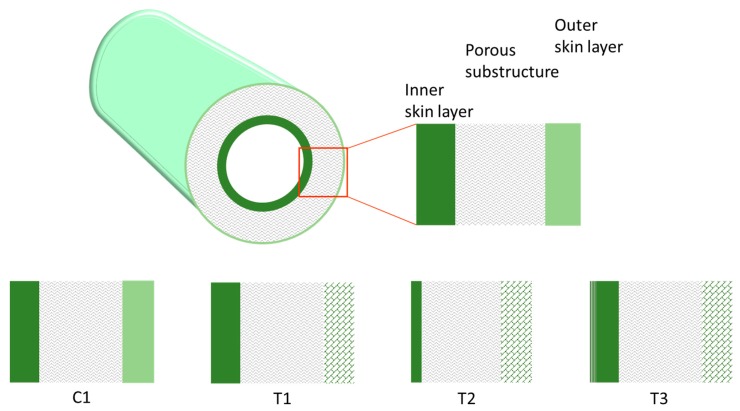
Scheme of the asymmetric structure for the investigated HFs.

**Figure 3:**
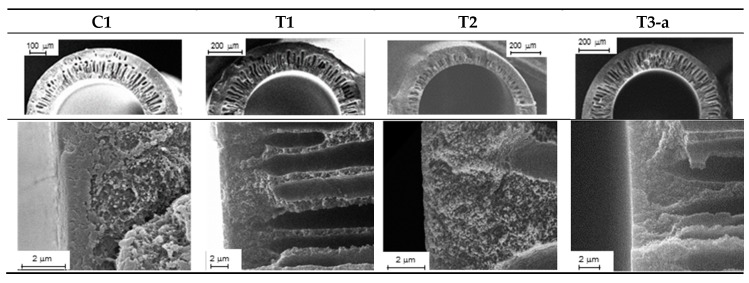
SEM images of the cross-section (up) and inner layer (down) for the investigated HFs.

Four types of HFs were produced that differed in their structure, as schematically depicted in Figure 2. A double skin layer was present in the C1 samples prepared by a conventional spinneret. Instead, the T1, T2, and T3 HFs, produced by the solvent-rich external fluid, had a porous external surface. Furthermore, the solvent addition to the BF led to thinner inner skin layers (T2), while the in-line crosslinking carried out for T3 HFs resulted in a different morphology, with a densified inner skin. The asymmetric structure of the investigated HFs is shown in Figure 3, evidencing the inner layer for each membrane type. Indeed, as discussed in Reference [4], the inner zone represents the major resistance to the gas transport. A thinner skin layer can be appreciated for T2 HFs with respect to C1 and T1 samples, owing to the use of a solvent-rich bore fluid. Furthermore, a denser structure was present in the T3-a HFs that were crosslinked.

The stability over time of the HFs was tested by performing successive gas permeation measurements, covering an aging time of ca. 30 months. This interval is compatible with the lifetime considered in the economic evaluations to size the membrane modules in gas separation. For this purpose, we used different permanent gases as molecular probes in order to relate the observed transport parameters to the evolution of the membrane microstructure. 

### 3.1. Long-Term Aging

**Figure 4:**
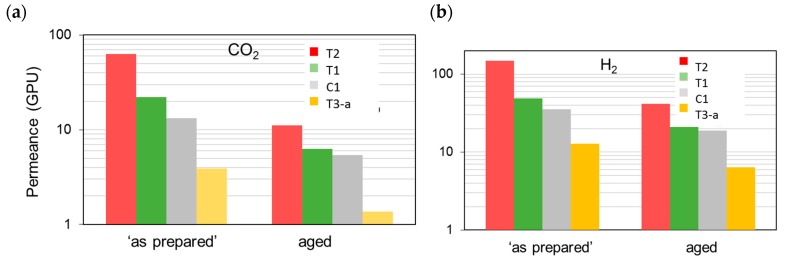
Permeance for the different HF samples tested “as prepared” and after long-term aging (30 months): (**a**) CO_2_; (**b**) H_2_.

**Figure 5:**
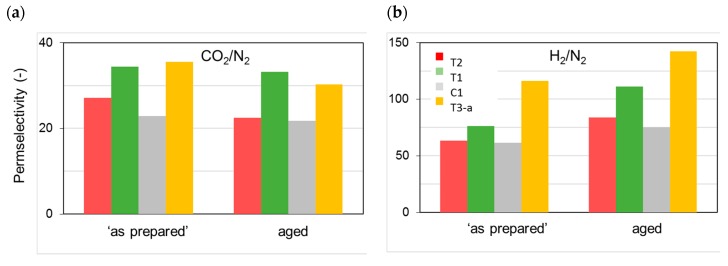
Permselectivity for the different HF samples tested “as prepared” and after long-term aging (30 months): (**a**) CO_2_/N_2_; (**b**) H_2_/N_2_.

Figure 4 reports the permeance measured on the membranes “as prepared” and aged for a long period (ca. 30 months) with reference to two species, CO_2_ and H_2_, of particular interest for industrial separations. These gases differ in their molecular dimensions and their polarizability. The first presents a high affinity for the polymer and is characterized by a good solubility, while the second is a small and fast gas. Considering the membranes “as prepared”, the permeance had the following order: T3 < the reference (C1) < T1 < T2 (see Figure 4). Permselectivity values proved the absence of pinholes (Figure 5) particularly for T1 with respect to C1 and T2; T3 instead presented the best size sieving behavior coupled with a low permeance owing to a “densified” inner skin layer.

All the HF samples, tested some months after their preparation, showed a reduction in the gas permeance. The observed permeance decline is related to the physical aging that is typical in glassy polymers and is accelerated in thin films [24]. This behavior originates from a relaxation of the polymer chains, which are in a state of non-equilibrium.

The resulting microstructure changes were reflected in the measured permeance changes, while the permselectivity also varied upon aging (Figure 5). Accordingly, the densification of the polymer matrix led to a reduced overall available free volume for permeation, whereas a different size distribution of the free volume elements was obtained. This can be inferred from the more marked size-sieving behavior and, thus, larger H_2_/N_2_ permselectivity values on the aged HFs (Figure 5b). Therefore, even after long-term aging, the membranes were defect-free and did not lose their selective properties. The initially observed order of gas permeation was unchanged over time.

The presented data demonstrate that the extent of the observed gas flux reduction depends on the sample morphology, which is strongly related to the preparation conditions (Figure 2). Indeed, the HFs spun in a conventional double orifice spinneret (C1) were unavoidably characterized by a double skin layer with an internal porous structure. When a solvent-enriched stream was added to the triple-orifice spinneret as external fluid, the external skin layer was destroyed and a fine porosity was present on the external side, while the main gas resistance was localized in the internal skin layer. If a solvent-enriched solution was used as both external and bore fluid, an even more important effect on gas permeance was observed (T1 [4]). However, the presence of an external fluid, independently of its composition, has a beneficial effect on the gas properties of the HFs. Indeed, these samples showed gas permeances higher than those prepared without an external fluid (C1). Similar considerations can be found concerning the permselectivity. The HFs prepared in the triple spinneret were more selective than the conventional C1 HFs, keeping these properties upon aging. 

### 3.2. Inner Crosslinking (T3 HFs)

In the case of crosslinked HFs, the decline in permeance was observed also with reference to the crosslinker agent concentration. Nevertheless, the gas permeance abatement upon aging in these membranes resulted less important than in the previous (C1, T1 and T2) samples.

**Figure 6:**
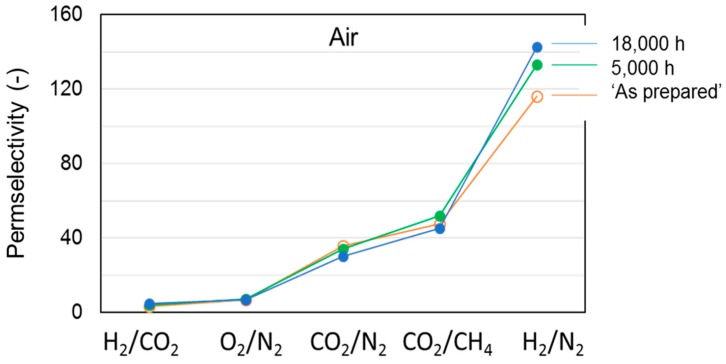
Effect of aging on permselectivity for different gas pairs in cross-linked HF membranes (T3-a).

**Figure 7:**
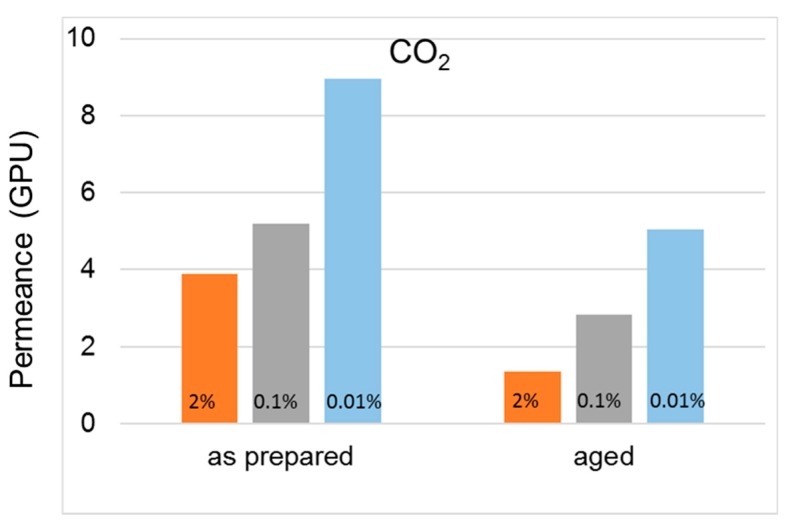
Change in CO_2_ permeance over time in cross-linked HF membranes (T3-type) prepared with different EDA concentrations in the bore fluid.

**Figure 8:**
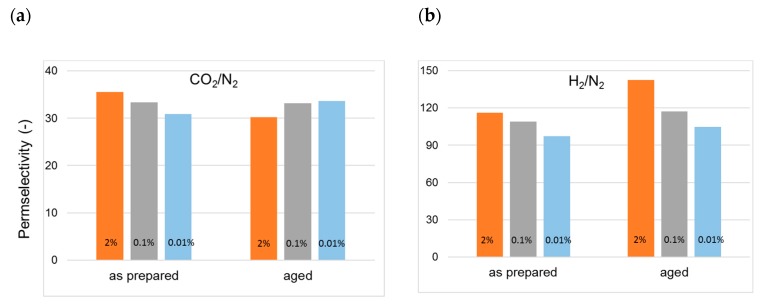
Change in permselectivity over time in cross-linked HF membranes (T3-type) prepared with different EDA concentrations in the bore fluid: (**a**) CO_2_/N_2_; (**b**) H_2_/N_2_.

A higher permselectivity was still more evident for the in-line crosslinked HFs (T3). As shown in Figure 4, the gas permeance of the inner crosslinked samples was depressed with respect to those prepared using pure water as BF. The diamino crosslinking enhanced certain selectivities, especially for gas pairs involving small molecules (Figure 5). Figure 6 shows, indeed, a larger permselectivity for a fast/slow gas pair such as H_2_/N_2_ in the samples prepared with the inner crosslinking and the solvent-rich EF (T3) if compared to the other HF samples. The aged T3-a membranes almost maintained the CO_2_/N_2_ permselectivity, with an increased H_2_/N_2_ (Figure 6). The greater part of the increase already occurred within the first 5000 h of aging. This happened even upon changing the crosslinker concentration in the range 0.01–2 wt %, as can be seen from Figure 7 and Figure 8. The results demonstrated that even a low amount of the crosslinker agent affected the HF microstructure.

In any case, the HFs obtained with an inner crosslinking increased their permselectivity upon aging and remained the most selective samples. Instead, those prepared in a triple-orifice spinneret using a solvent-solution as BF (T2), even after the aging, were the most permeable, showing a gain in permselectivity when smaller gases were involved.

### 3.3. Time Evolution 

**Figure 9:**
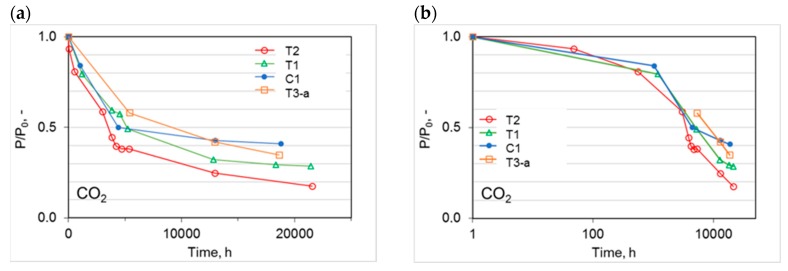
Time evolution of the CO_2_ permeance relative to the initial value (*P*_0_) for the different HF samples: (**a**) linear plot; (**b**) semi-log plot.

Due to the progressive nature of the physical aging, it becomes important to follow the time evolution for the membrane performance and not only the values attained after long periods. Therefore, Figure 9a illustrates the CO_2_ permeation flux as a function of time for the prepared HFs. The observed trend was a non-linear decay in agreement with a “self-retarding” model for the aging process, as proposed by Struik [25,26]. The gas permeation flux declined significantly in the first six months (ca. 4300 h) after the spinning. Then, the decay rate was reduced. A clear leveling off can be seen for the C1 and T1 HFs, suggesting the attainment of an “equilibrium situation”. Both HF types were prepared in the conventional spinneret or in the triple-orifice spinneret using water as bore fluid. On the contrary, the most permeable (T2) and the least permeable (T3) HFs tended to change their properties even after a long aging time with different rates. 

A larger initial permeance resulted in a greater relative drop (Figure 9a). Thus, the more permeable samples (T2), where a solvent-enriched solution was used as both internal and external fluid, had a larger reduction in the relative permeance. Nevertheless, these samples remained the most permeable. Instead, the crosslinked HFs (T3), at similar aging times, displayed a less marked permeance decline over time because their matrix was partially restricted by the formed connections between the polymer chains. This is in agreement with other studies on UV crosslinked thin films [27]. In general, crosslinking is one of the effective ways of slowing down the physical aging [28,29].

Upon plotting the normalized CO_2_ permeation data on a logarithmic scale for the time abscissa (Figure 9b), the shape of the curves changed and could be compared to those proposed by Rowe et al. [17]. By analyzing the CO_2_ permeation over time in the semi-log plot, the peculiar behavior of thin polymer films could be appreciated. In particular, this trend was the same reported for thin films with a thickness of ca. 500 nm based on polysulfone and Matrimid [17], differing from thicker films (bulk) that presented an almost linear drop with a smaller slope. Instead, very thin films of Matrimid (ca. 50 nm or less) displayed a single slope for the permeability decay [17].

**Figure 10:**
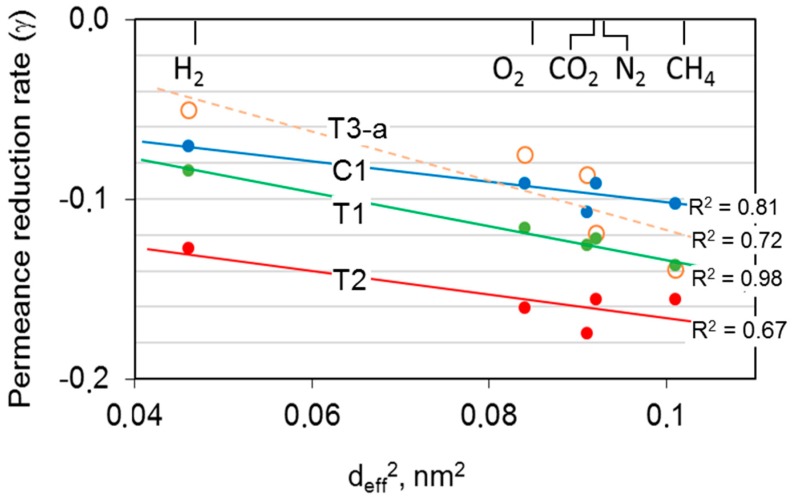
Decay of the gas permeance as a function of the squared gas molecular diameter for the different HF samples.

The above-reported results indicate a chain rearrangement in the HF structure with a reduction in the free volume available for the permeation. The tests carried out with different gases were helpful to gain more insight into the free volume size distribution within the polymer matrix. Indeed, the accessible free volume depends on the gas probe molecular dimension [30,31]. According to the solution diffusion mechanism for the gas transport through polymeric films [32], the gas transport process in glassy polymers is mainly controlled by the diffusion process, rather than by the sorption. Diffusion coefficients can be correlated to the square of the gas molecular size [33]. For this reason, it can be useful to correlate the logarithm of permeance decay over time to the molecular diameter of gas, according to the literature [16]. Figure 10 reports the gas permeance reduction rate, defined as (γ = $\frac{\partial \;lnP}{\partial \;lnt}$), calculated on the basis of the long-term aging data. The data showed linearity between log*P* and log*t* [8]. A similar slope can be seen for the uncrosslinked HFs, while the crosslinked T3 membranes displayed a lower influence of the aging on the permeance.

Typically, a larger gas molecule presented a greater observed permeance decay. Therefore, the novel size distribution for the free volume elements in the aged polymeric matrix provided a greater resistance to the transport of bulkier molecules such as CH_4_. A perfect agreement for T1 and T3 samples was observed. On the contrary, in the case of C1 and T2 samples, the correlation was not equally good, providing a less regular trend with all permanent gases. This can be explained in terms of contribution to the transport by the porous substrate of the hollow fibers in C1 and T2 samples. In the microporous substructure, the gas transport followed a Knudsen mechanism and, thus, the gas permeance was inversely proportional to the square root of the molecular weight rather than to the molecular size. Indeed, these samples showed selectivity values lower than the intrinsic values of the polyimide for different reasons. In the case of C1 HFs, the presence of two skins not equally selective caused the lower selectivity, whereas the extreme thinness of the single inner skin was the reason for reduced selectivity in the T2 sample with respect to T1. 

### 3.4. Effect of the Post-Treatment

All four types of hollow fibers analyzed in the present work were tested without any post-treatment after the preparation, with a conclusive drying in air after the washing step in water. However, almost all HFs considered in the literature were subjected to a final drying step from water by using a solvent exchange protocol, finalized to keep open the porous substructure of the samples. For this reason, we performed a customized solvent exchange protocol on the HFs that showed depleted physical aging in ca. 30 months (C1 and T1 samples). These HFs resulted also structurally the more robust. Successive baths in alcohol were used for the solvent exchange after spinning. 

### 3.5. Conventional Spinneret (C1 HFs)

**Figure 11:**
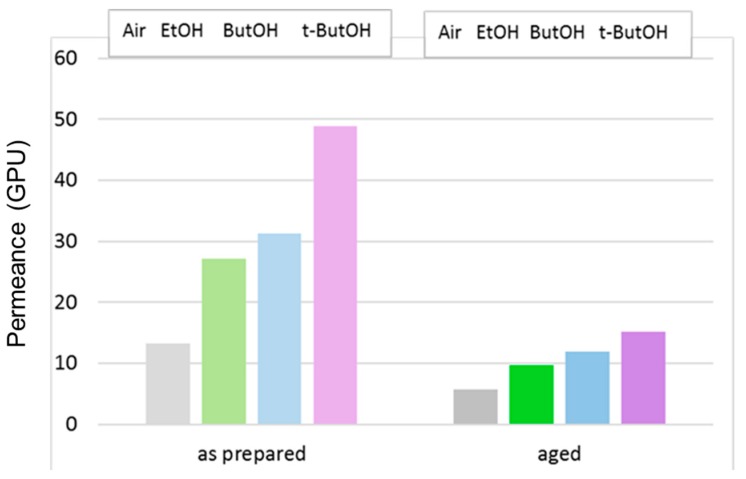
CO_2_ permeance in C1 membranes that were dried in air or solvent-exchanged. Tests carried out just after the preparation or after long-term aging.

The effect of the post-treatment on the C1 HFs prepared with the conventional spinneret is highlighted in Figure 11, reporting the data of CO_2_ permeance for membranes tested just after the post-treatment or after long-term aging.

The post-treatment is usually performed in order to keep the porous substructure unchanged in asymmetric membranes. However, it can be clearly seen that the gas permeance was also reduced on the post-treated HFs, to an even greater extent than those dried directly from water. This behavior was also observed in the case of superglassy Polymers of Intrinsic Microporosity (PIMs) treated with lower alcohols to remove the residual casting solvent [34].

In any case, the gas permeance order was preserved upon aging; a larger alcohol molar volume resulted in a greater permeance gain for the treated membranes with respect to drying in air.

**Figure 12:**
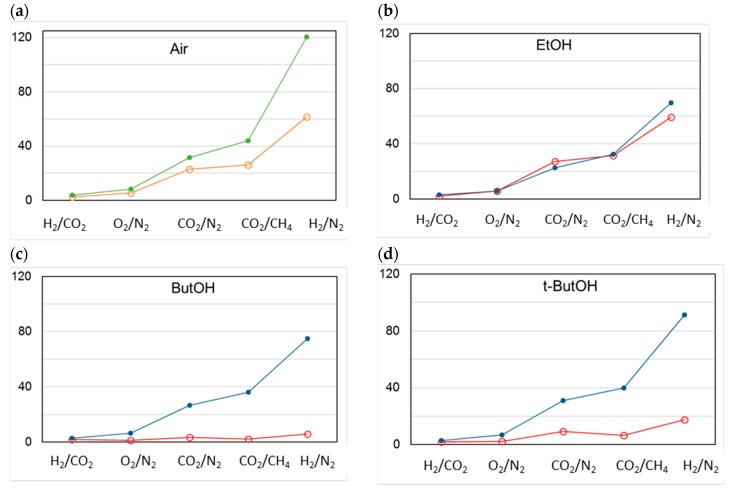
Change in permselectivity for different gas pairs in C1 membranes post-treated according to different protocols. Open circles: “as prepared” HFs; closed circles: aged HFs.

Figure 12 shows the change in different permselectivities upon aging, in the case of membranes C1 dried in air (Figure 12a) or according to a solvent exchange protocol (Figure 12b–d). The permselectivity increased upon aging in any case in the order H_2_/CO_2_ < O_2_/N_2_ < CO_2_/N_2_ < CO_2_/CH_4_ < H_2_/N_2_. The HFs dried in air and aged were the most selective samples, while, for the HFs dried from EtOH, no permselectivity difference over time was observed. Interestingly, the samples treated with larger alcohols (ButOH and *t*-ButOH) recovered their permselectivity upon aging.

### 3.6. Triple-Orifice Spinneret (T1 HFs)

**Figure 13:**
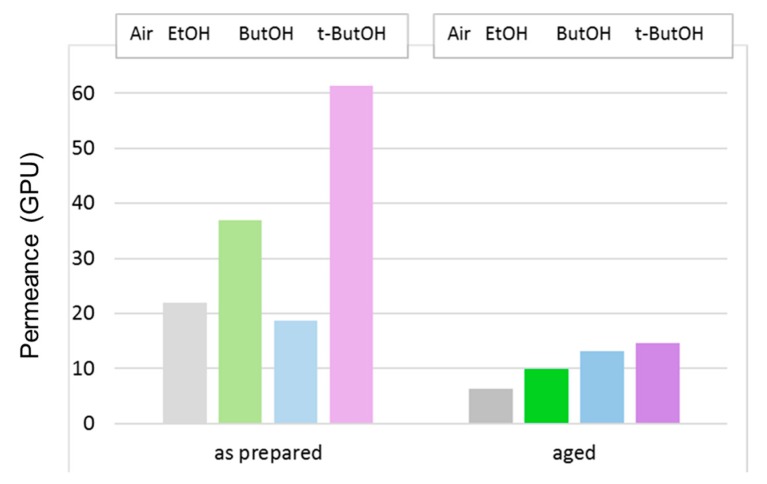
CO_2_ permeance (GPU) in T1 membranes that were dried in air or solvent-exchanged. Tests carried out just after the preparation or after long-term aging.

The effect of the post-treatment on the HFs prepared with the triple-orifice spinneret, feeding a solvent-rich EF (T1), is highlighted in Figure 13, in which the data of CO_2_ permeance are reported for membranes tested just after the post-treatment or after long-term aging. 

On the “as prepared” T1 HFs, all alcohols except for the larger ButOH were effective in increasing the gas permeance. Upon aging, a larger alcohol molar volume resulted in a larger final permeance. Despite the larger CO_2_ permeance decline compared to the HF samples prepared by conventional spinneret, HFs spun by triple-orifice spinneret remained the most permeable upon aging.

**Figure 14:**
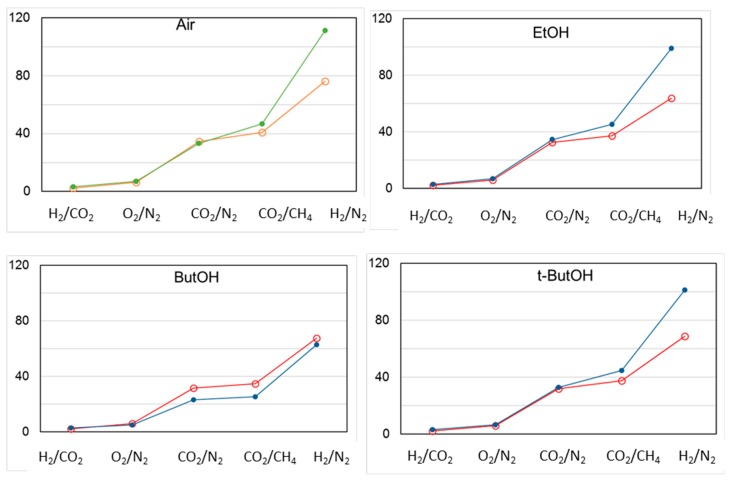
Change in permselectivity for different gas pairs in T1 membranes post-treated according to different protocols. Open circles: “as prepared” HFs; closed circles: aged HFs.

The permselectivity remained constant over time for all gas pairs considered, independently of the type of alcohol used for the drying step, except for H_2_/N_2_. In this case, a comparable increase upon aging was observed (Figure 14). *t*-ButOH produced more interesting results in terms of both gas permeance and permselectivity upon aging, as already proven in a previous study [6]. Indeed, the globular shape of *t*-butanol is capable of enlarging the polymer matrix with respect to smaller alcohols. In addition, *t*-butanol has a low polarity and close solubility parameters to Matrimid^®^ [6]. On the other hand, 1-butanol was not efficient as *t*-butanol in increasing the permeation flux of the membrane, even if they are isomers. This trend can be related to the linear shape of the 1-butanol molecule, allowing its easy penetration inside the Matrimid polymer matrix, with a lower effect on the polymer chain arrangement. In general, the molecular shape of a penetrant is the key factor in determining the diffusion coefficients within a polymer matrix (e.g., gases). For this reason, the diffusion process is easier in the case of a linear molecule such as CO_2_ vs. the bulkier CH_4_.

## 4. Conclusions

In the present work, long-term permeation tests with different gases on asymmetric polyimide HFs, prepared by the dry-jet/wet-quench spinning, were performed. 

The physical aging of the material resulted in densified skin layers; thus, it depressed the permeation rates but enhanced the permselectivity for fast/slow gases. In general, a self-retarding behavior was found by analyzing the time evolution of the gas permeance. The most important decline was registered in the first aging period, within the first six months. Then, a partial softening of the slope was observed. According to the specific preparation mode, the HF samples aged differently. The crosslinked membranes were more stable over time. A thinner effective thickness of the skin layer resulted in a stiffer gas permeance decline over time. Despite the largest permeance reduction, the HFs spun in the triple-orifice spinneret remained the most permeable samples.

The increase in permeance due to the treatment with alcohol gradually decreased over time. However, the gas permselectivity was maintained and, in some cases, even increased.

## Figures and Tables

**Table 1:** Composition (weight basis) of bore fluid (BF) and external fluid (EF) for the preparation of the HF batches. NMP—*N*-methyl-2-pyrrolidone; EDA—ethylene diamine.

HF Code	Spinneret	BF	EF	Crosslinking
**C1**	Conventional	Water	–	–
**T1**	Triple	Water	NMP/water (95/5)	–
**T2**		NMP/water (60/40)	NMP/water (95/5)	–
**T3-a**		EDA in water (2%)	NMP/water (95/5)	Internal
**T3-b**		EDA in water (0.1%)	NMP/water (95/5)	Internal
**T3-c**		EDA in water (0.01%)	NMP/water (95/5)	Internal
